# Ivabradine: A Potential Therapeutic for Children With Refractory SVT

**DOI:** 10.3389/fcvm.2021.660855

**Published:** 2021-08-03

**Authors:** Nour K. Younis, Bernard Abi-Saleh, Farah Al Amin, Omar El Sedawi, Christelle Tayeh, Fadi Bitar, Mariam Arabi

**Affiliations:** ^1^Faculty of Medicine, American University of Beirut Medical Center, Beirut, Lebanon; ^2^Internal Medicine Department, Division of Cardiology, American University of Beirut Medical Center, Beirut, Lebanon; ^3^Pediatric Department, Division of Pediatric Cardiology, American University of Beirut Medical Center, Beirut, Lebanon

**Keywords:** ivabradine, pediatric patients, SVT, supraventicular tachycardia, tachyarhythmia

## Abstract

**Background:** In April 2015, ivabradine was approved by the Food and Drug Administration for the treatment of patients with coronary artery disease and heart failure (HF). The use of this medication has been linked with improved clinical outcomes and reduced rates of hospitalization in patients with symptomatic HF and a baseline heart rate of 70 bpm and above. Nonetheless, little is known about the use of ivabradine in pediatric patients with supraventricular tachycardia (SVT). This use is not well-studied and is only endorsed by a few case reports and case series.

**Aim:** This study discusses the off-label utilization of ivabradine in pediatric patients with SVT, and highlights its efficacy in treating treatment-resistant (refractory) SVT.

**Methods:** We conducted a retrospective single-center observational study involving pediatric patients with SVT treated at our center between January 2016 and October 2020. We identified the total number of patients with SVT, and the number of patients with refractory SVT treated with Ivabradine. Similarly, we performed a thorough review of the databases of PubMed, Medline and Google Scholar to compare the clinical course of our patients to those described in the literature.

**Results:** Between January 2016 and October 2020, 79 pediatric patients with SVT were seen and treated at our center. A treatment-resistant SVT was noted only in three patients (4%). Ivabradine was used in these patients as a single or combined therapy. The rest (96%) were successfully treated with conventional anti-arrhythmics such as β-blockers, flecainide, and other approved medications. In the ivabradine group, successful reversal to sinus rhythm was achieved in two of the three patients (66%), one patient was treated with a combination therapy of amiodarone and ivabradine, and the other patient was treated only with ivabradine.

**Conclusion:** Overall, promissory results are associated with the use of ivabradine in pediatric patients with refractory SVT. Ivabradine appears to be a safe and well-tolerated medication that can induce adequate suppression of SVT, complete reversal to sinus rhythm, and effective enhancement of left ventricular function.

## Introduction

Ivabradine is a novel antiarrhythmic medication that was recently approved, on April 2015, by the food and drug administration (FDA) for the treatment of stable angina and systolic heart failure (HF) (1, 2). It was first introduced as an anti-anginal medicine by the European Medicines Agency (EMA) in 2005 and then as a heart failure medicine in 2012 (2). Ivabradine is a heart rate-reducing medication that was shown to improve overall morbidity and mortality in coronary artery disease (CAD) and HF patients, respectively (3). It is now considered an alternative to conventional therapies and is used particularly in cases where β-blockers are either ineffective or contraindicated (3, 4).

The use of this anti-arrhythmic in pediatric patients with supraventricular tachycardia (SVT) is still not well-endorsed. To date, the off-label utilization of ivabradine in these patients has been grasped only by a few case reports and case series. Nevertheless, the promissory results reported by these studies have encouraged this off-label use in particular cases that are refractory to well-studied formerly approved antiarrhythmics such as β-blockers, flecainide, and amiodarone among others. Indeed, the paucity of published data supporting this use should prompt physicians to share their experience with this medication in not well-studied populations. Interestingly, shared experience can guide the use of this medication. It can also support the establishment of higher-quality randomized clinical trials that compare ivabradine to well-known therapies.

This retrospective study aims to highlight the efficacy of ivabradine in reversing pediatric SVT and preventing life-threatening associated events such as tachycardia-induced cardiomyopathy. Additionally, it aspires to provide a review of the current evidence that discusses the use of this novel medication not only in pediatric patients but also in adults.

## Mechanism of Action of Ivabradine

**Figure 1:**
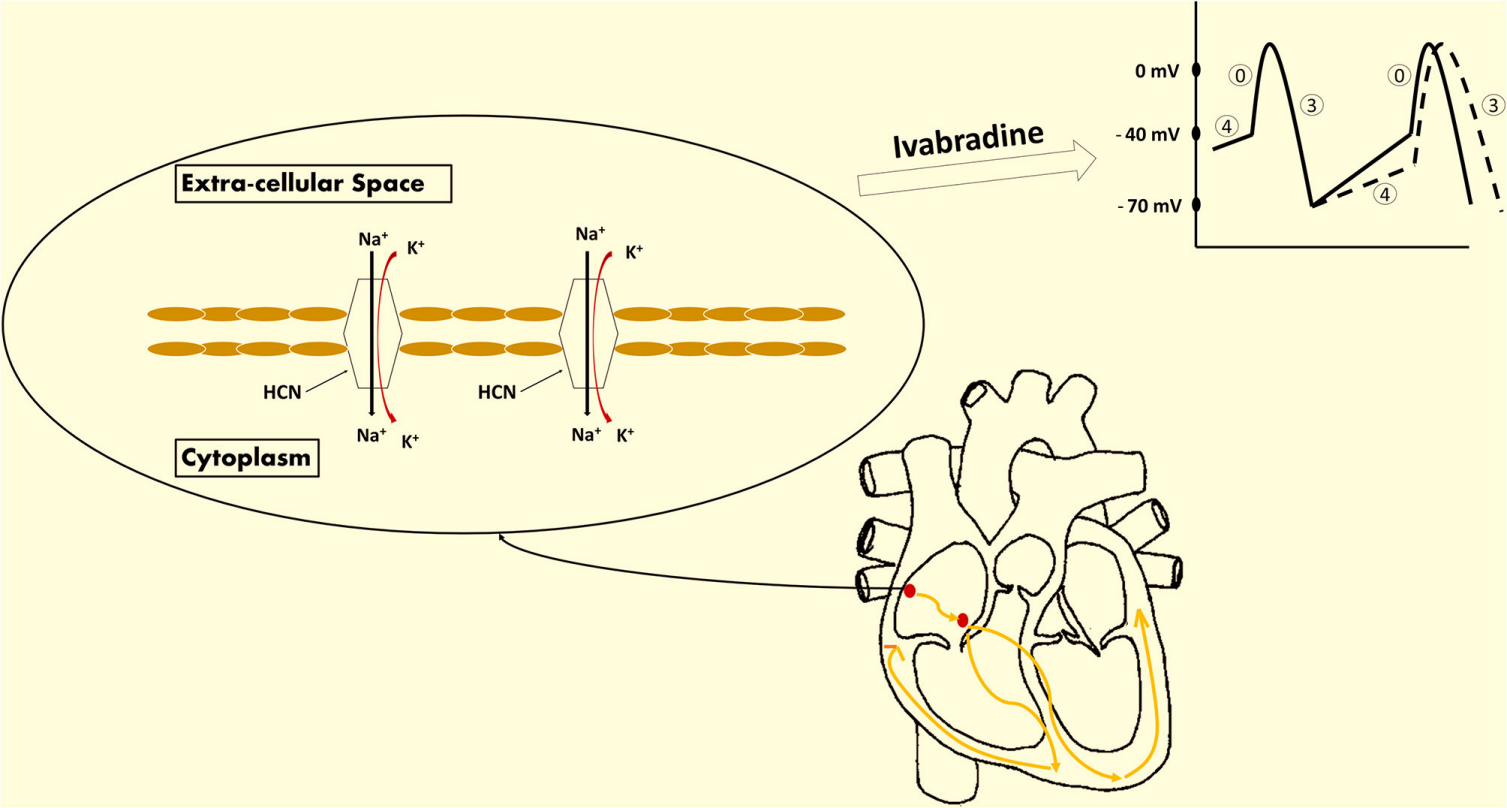
Ivabradine's mechanism of action. Ivabradine is a novel medication approved for the treatment of coronary artery disease (CAD) and heart failure (HF). It exhibits a negative chronotropic effect induced by the selective inhibition of the funny current channels of the sinoatrial (SA) node. The funny current is a mixed inward current of sodium and potassium that passes through the hyperpolarization-activated cyclic nucleotide-gated (HCN) channels. This current is essential for the spontaneous depolarization of the pacemaker cells of the SA node. The extent of HCN channels activation is reflected by the slope of phase 4. As a result, ivabradine exhibits its heart rate-reducing effect via the inhibition of these channels and the deceleration of phase 4 depolarization (dashed lines reflect the effect induced by ivabradine on the action potential of the SA node).

Ivabradine is a rate-reducing medication that exerts its negative chronotropic effect through selective inhibition of the funny current of the sinoatrial node (SA node), as shown in Figure 1. It contributes subsequently in improving diastolic filling and ventricular functioning (2–5). The funny current represents a mixed inward current of sodium and potassium, caused by the activation of the hyperpolarization-activated cyclic nucleotide-gated (HCN) channels (6, 7). This current is primarily regulated by the autonomic nervous system (ANS) mediated by intracellular cAMP, and also by membrane hyperpolarization during diastole (6). There are 4 isoforms of HCN denoted by HCN1, HCN2, HCN3, and HCN4, respectively, with the latter being the most heavily expressed channel in the sinus node (6, 8, 9).

HCN4 represents the prime player that mediates the generation of the SA funny current (9, 10). In the light of this, ivabradine is the only available pure inhibitor of HCN4 employed in the regulation of heart rate in patients with coronary artery disease and heart failure, respectively (8, 11, 12). Ivabradine displays use-dependence properties, and is thus maximally effective at increased heart rates (12–14). Additionally, ivabradine exhibits no ionotropic properties (see Table 1). It exerts no effect on cardiac contraction and relaxation (2–5). Ivabradine has no effect on vascular resistance as well (12, 14, 15). To this end, as compared to β-blockers, ivabradine can be safely administered to patients with acute decompensated systolic heart failure by virtue of its neutral ionotropic effect (16). Nonetheless, further studies are definitely needed to approve the use of this medication among these patients.

**Table 1:** Cardiac effects of Ivabradine along with its mechanism of action and clinical uses.

**Ivabradine**	
FDA approval	April 2015
Mechanism of action	Selective inhibition of the funny current of the sinoatrial node
Effects exerted on cardiac functions	Negative Chronotropic effect No ionotropic or lusitropic effects
Indications of use	Coronary artery disease Heart failure
Off-label use	Tachyarrhythmias (i.e., inappropriate sinus tachycardia, supraventricular tachycardia)

## Methods

### Study Design and Population

Our study involves a retrospective observational analysis of the medical records of pediatric patients with supraventricular tachycardia treated at the Children's Heart Center at the American University of Beirut-Medical Center (AUBMC) between January 2016 and October 2020, inclusive. It includes all patients aged 18 years and below diagnosed with the following SVT: atrial tachycardia, atrioventricular reciprocating tachycardia (AVRT), atrioventricular nodal re-entrant tachycardia (AVNRT), junctional ectopic tachycardia, Wolff-Parkinson White syndrome (WPW), and unspecified SVT. On the contrary, it excludes patients who (1) presented prior to January 2016, (2) are aged more than 18 years at the time of diagnosis, or (3) are not known to have SVT.

### Data Collection and Analysis

After obtaining Institutional Review Board approval, we collected data from the hospital medical records, and analyzed the following parameters: demographic information, primary diagnosis, past medical history, past surgical history, provided primary medical therapy, concomitant medical therapies, and clinical outcomes. We reviewed as well the electrocardiogram (ECG) and the echocardiography of the studied patients.

We determined the total number of eligible patients treated at our tertiary care center during the afore-mentioned period, after the introduction of ivabradine into the Lebanese pharmaceutical market. We reviewed thoroughly the parameters of interest and identified the patients with refractory SVT who were treated with ivabradine. This article provides a comprehensive description of these cases' clinical presentation and their response to the distinct medical therapies. Moreover, the clinical course of these patients and their response to ivabradine are compared to the cases reported in the literature.

### Literature Review

In this study, we aspire as well to provide an updated review about ivabradine and its use in treating SVT among other cardiovascular diseases. A search of the relevant and valid articles published in the English language has been performed and concluded by June 2021. Articles were retrieved from the following databases: PubMed and Medline, and Google Scholar. Articles in which ivabradine was used as a treatment for cardiovascular diseases including SVT, heart failure and coronary artery disease, were reviewed. We searched for the following MeSH terms and keywords: ivabradine AND [(atrial tachycardia, atrioventricular reciprocating tachycardia (AVRT), atrioventricular nodal re-entrant tachycardia (AVNRT), junctional ectopic tachycardia, supraventricular tachycardia, or WPW] OR (heart failure) OR (Coronary artery disease, coronary disease, CAD, angina, stable angina, unstable angina, microvascular angina, or angina pectoris). We excluded review articles, letters to the editor and comments. We limited the search to pediatric patients aged up to 18 years. Yet, we reviewed the articles in which adult patients were included as well. We imposed no limits on the publication date and the country of origin.

Twenty studies were retrieved, and only 16 studies were relevant to our study (see Table 2). One study was about the use of ivabradine in treating children with dilated cardiomyopathy (DCM) and symptomatic heart failure. The remaining studies were about the use of this medication in children with tachyarrhythmias.

**Table 2:** This table summarizes the retrieved articles that are relevant to our study along with their types and references.

**#**	**Retrieved relevant studies**	**Study type**	**References**
1	Clinical and Electrophysiological Correlates of Incessant Ivabradine-Sensitive Atrial Tachycardia	Prospective Observational Study	(17)
2	Reversal of tachycardiomyopathy due to left atrial tachycardia by ivabradine.	Case report	(18)
3	A case of atrial tachycardia treated with ivabradine as bridge to ablation.	Case report	(19)
4	Potential new indication for ivabradine: treatment of a patient with congenital junctional ectopic tachycardia.	Case report	(20)
5	Adjunctive ivabradine in combination with amiodarone: a novel therapy for pediatric congenital junctional ectopic tachycardia	Case series	(21)
6	Ivabradine for junctional ectopic tachycardia in post congenital heart surgery.	Case series	(22)
7	Ivabradine is an effective antiarrhythmic therapy for congenital junctional ectopic tachycardia-induced cardiomyopathy during infancy: case studies.	Case series	(23)
8	A pediatric case of cardiomyopathy induced by inappropriate sinus tachycardia: efficacy of ivabradine.	Case report	(24)
9	Ivabradine in postural orthostatic tachycardia syndrome: preliminary experience in children.	Retrospective observational study	(25)
10	Ivabradine in children with dilated cardiomyopathy and symptomatic chronic heart failure.	Randomized clinical trial	(26)
11	Ivabradine in post-operative junctional ectopic tachycardia (JET): breaking new ground	Retrospective observational study	(27)
12	Ivabradine as an adjunct for refractory junctional ectopic tachycardia following pediatric cardiac surgery: a preliminary study	Retrospective observational study	(28)
13	Use of ivabradine for the treatment of congenital junctional ectopic tachycardia	Case series	(29)
14	Ivabradine as a stabilizing anti-arrhythmic agent for multifocal atrial tachycardia	Case report	(30)
15	Ivabradine for treatment of tachyarrhythmias in children and young adults	Case report	(31)
16	Ectopic atrial tachycardia in a 12-month-old girl treated with ivabradine and beta-blocker, a case report	Case report	(32)

Our study aims to assess the efficacy of ivabradine in treating pediatric patients with supraventricular tachycardia. Hence, we will discuss the latest evidence derived from high-quality clinical studies that include adult and pediatric patients with heart failure and coronary artery disease. Nonetheless, the core discussion is going to concentrate on the most pertinent studies that involve pediatric patients with supraventricular tachycardia.

## Results

Seventy-nine pediatric patients with SVT presented to our center between January 2016 and October 2020. We treated the majority (96%) of these patients with conventional therapies such as β-blockers, flecainide, and amiodarone. In our center, β-blockers are considered first-line pharmacological therapy in all patients with non-operative SVT. Flecainide comes second in patients with an anatomically normal heart. Amiodarone is provided primarily to patients with post-operative SVT or treatment-resistant SVT. Only three patients (4%) were deemed with SVT refractory to these standard medications and were started on ivabradine which was considered the last resort pharmacological therapy prior to surgical ablation. Herein, we discuss the off-label use of ivabradine in these patients and its efficacy in reverting their SVT into sinus rhythm.

### Cases Presentation

#### Case 1

A 2-year-6-month-old girl was transferred to our hospital from a primary care center for the management of tachyarrhythmia. Eight months prior to her presentation, she was found to have an increased heart rate (HR) of around 200 bpm on routine physical examination. At that time she was asymptomatic and her echocardiography was normal. Nevertheless, a week prior to her presentation to our center, an increased HR of 190 bpm was noted again on routine exam. Repeat echocardiography revealed LV dilatation along with a reduced LV systolic ejection fraction of 35%. ECG showed supraventricular tachycardia (SVT), namely atrial tachycardia. She failed a trial of adenosine and responded weakly to metoprolol. The patient was discharged on metoprolol 25 mg/day divided into two doses. She presented 2 days later to the same hospital. She had a HR of 250 bpm. She received two doses of adenosine, one dose of amiodarone and several doses of flecainide during her admission. However, no improvement was noted after 5 days of treatment, and also after direct current cardioversion. She was then transferred to our center for adequate rate control and appropriate investigation.

**Figure 2:**
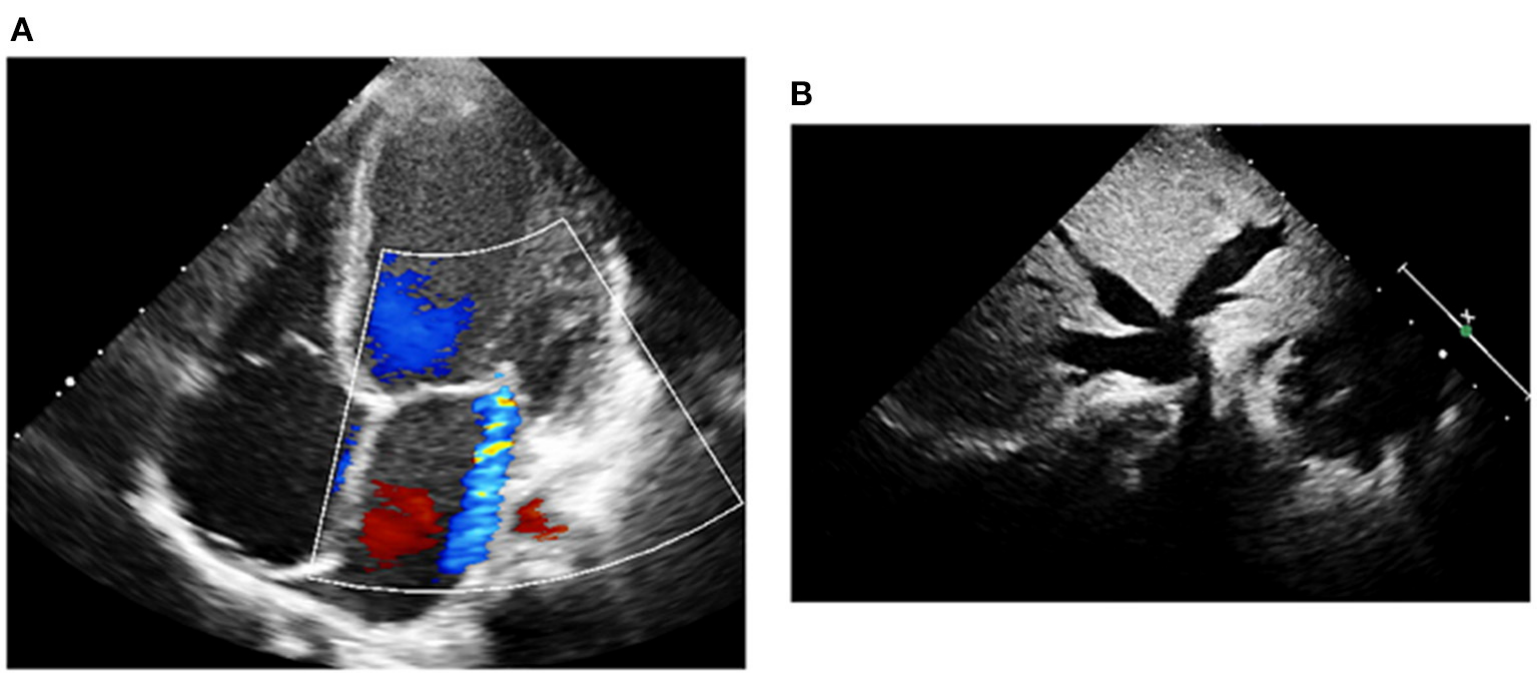
Echocardiography of patient #1 prior to treatment with ivabradine. **(A)** A moderate mitral regurgitation and a severely dilated left ventricle were observed on echocardiography. **(B)** Liver ultrasound revealed dilated inferior vena cava and hepatic veins.

**Figure 3:**
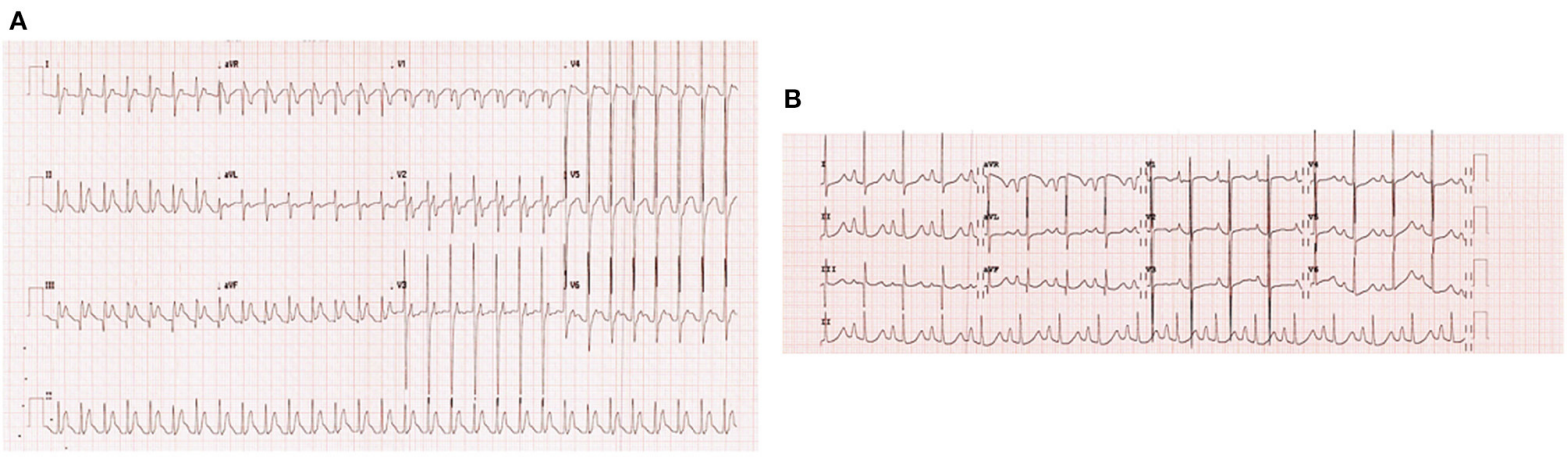
ECGs of patient #1 pre- and post-ivabradine. **(A)** ECG revealing atrial tachycardia. **(B)** ECG revealing sinus rhythm (ECG speed: 25 mm/s, and sensitivity: 10 mm/mv). As discussed, this patient was successfully treated with ivabradine, she reverted from atrial tachycardia to sinus rhythm after receiving an adequate course of ivabradine.

Upon admission to our PICU, HR was 190 bpm. On examination, she was pale, tachypneic and hypoactive, but responsive to verbal stimulation. A third heart sound was noted on cardiac auscultation. Additionally, she had cool extremities and palpable hepatomegaly. Echocardiography was repeated, it showed worsened LV dilatation and severe LV systolic dysfunction (EF was around 20%), and moderate mitral regurgitation (see Figure 2). ECG revealed an atrial tachycardic rhythm. Patient was given adenosine. She received a second higher dose of adenosine and an infusion of amiodarone. She failed to respond adequately to these medications. Consequently, the patient was started on ivabradine at 0.15 mg/kg/day. An adequate response was noted few hours after the first dose of ivabradine. HR decreased to 90 bpm and she reverted to sinus rhythm. Figure 3 shows the patient's ECG pre- and post-ivabradine. The dose of ivabradine was adjusted to 0.033 mg/kg thrice daily. Adequate response was noted and the patient was discharged on ivabradine.

One month after the discharge, she presented to our clinic and was found to have a normocardic sinus rhythm on ECG, and an improved cardiac function on echocardiography (EF of 50%). In short, this is a case of SVT-induced DCM managed appropriately with ivabradine.

#### Case 2

A male newborn was transferred to our tertiary hospital from a primary hospital in rural areas for surgical treatment of suspected truncus arteriosus. Physical exam was pertinent for decreased oxygen saturation of 80%, dysmorphic facial features, grade III systolic murmur and faint femoral pulses. Echocardiography was significant for truncus arteriosus Edward's type I, truncus valve regurgitation, stenotic right pulmonary artery (RPA), and aberrant coronary and right subclavian arteries.

**Figure 4:**
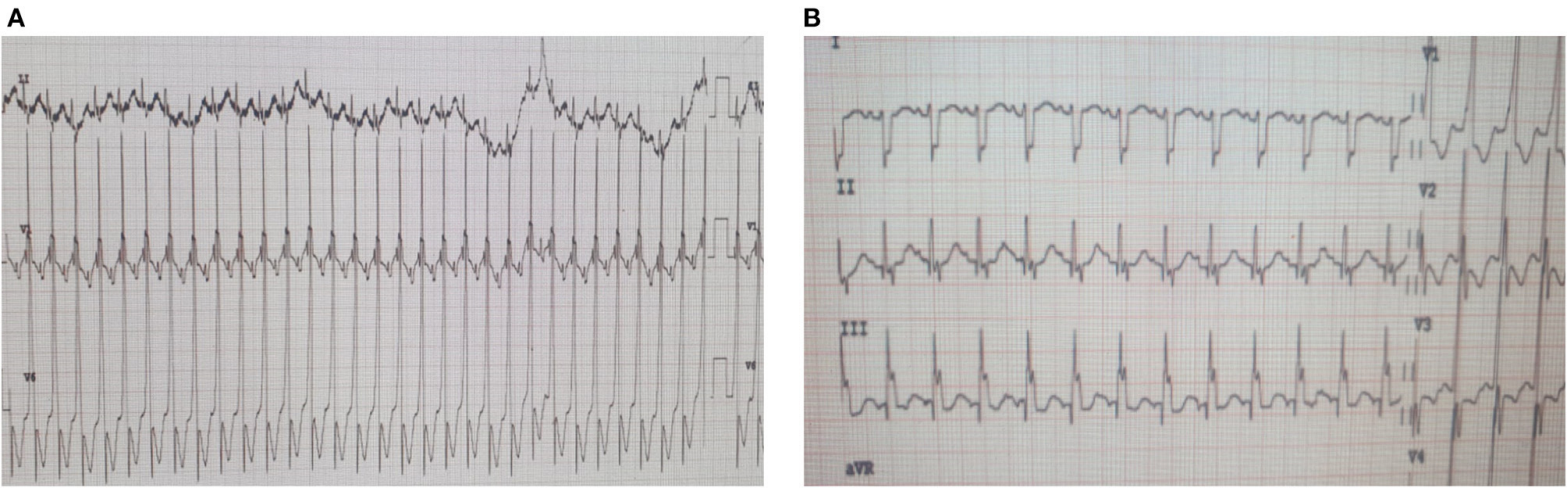
ECGs of patient #2 pre- and post-ivabradine. **(A)** ECG revealing atrial tachycardia. **(B)** ECG revealing sinus rhythm (ECG speed: 25 mm/s, and sensitivity: 10 mm/mv). This patient was reverted adequately to sinus rhythm with combined amiodarone and ivabradine.

Few days after admission, he underwent total surgical repair of his anomaly and dilatation of RPA. His clinical course remained uncomplicated until post-operative day 9 when he developed incessant tachycardia reaching 195 bpm. ECG was consistent with atrial tachycardia (Figure 4). Despite receiving an adequate dose of amiodarone, the patient's condition persisted. As a result, we added ivabradine at a dosage of 0.05 mg/kg/day divided into two doses. On the next day, the dosage was increased to 0.075 mg/kg/day. He reverted successfully to sinus rhythm, and had no side effects such as hypotension or bradycardia.

This is a case of atrial tachycardia successfully treated with a combined regimen of amiodarone and ivabradine.

#### Case 3

A 9-year-old female, with a known medical history of severe dilated cardiomyopathy secondary to ectopic atrial tachycardia, presented to our clinic for a regular check-up. Echocardiography was significant for severe left ventricular (LV) dilatation, markedly reduced LV systolic function reflected by an ejection fraction of 30%, and severe mitral regurgitation. ECG revealed a regular sinus rhythm. Despite being on several medications including aspirin, carvedilol, digoxin, enalapril, and spironolactone, the patient's cardiac condition failed to improve. A gradual worsening in the systolic function of LV and the degree of mitral regurgitation was noted over the last few visits.

Upon follow up, echocardiography findings were similar to the previous findings. However, ECG was significant for ectopic atrial tachycardia. We placed a 24-h Holter monitor a week after to evaluate appropriately her heart rhythm. Holter revealed an incessant atrial tachycardia interrupted by a few sinus beats.

**Figure 5:**
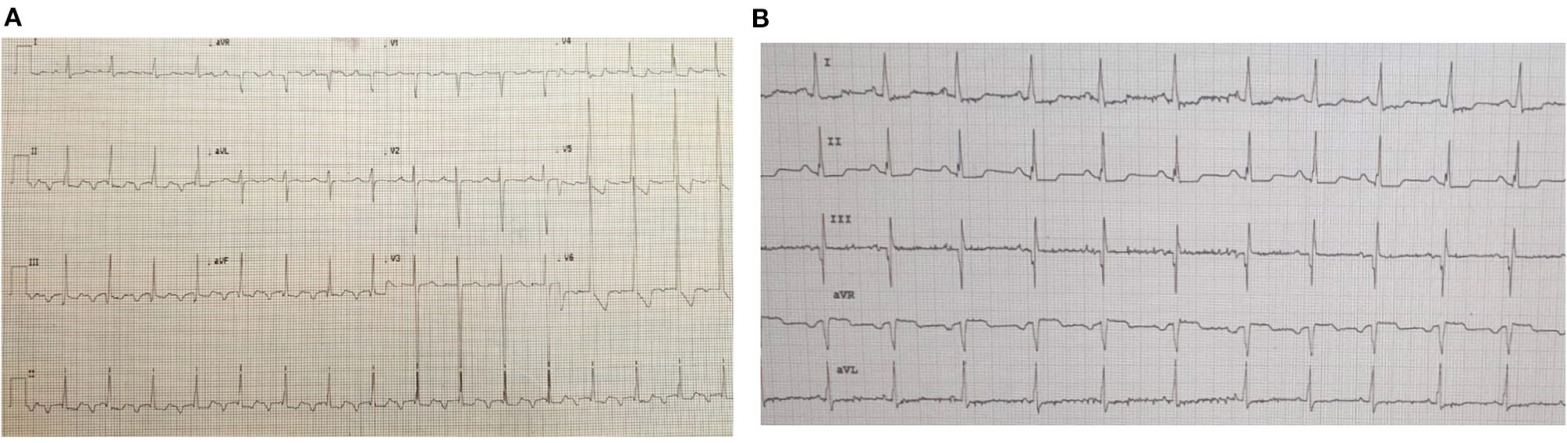
ECGs of patient #3 pre- and post-ivabradine. **(A)** ECG showing incessant atrial tachycardia. **(B)** ECG showing sinus rhythm few hours after ivabradine initiation (ECG speed: 25 mm/s, and sensitivity: 10 mm/mv). This initial response to ivabradine was not maintained. As a result, the patient was switched to amiodarone.

Consequently, the patient was admitted to the pediatric intensive care unit (PICU) for observation and initiation of appropriate anti-arrhythmic medications. Upon admission, carvedilol and digoxin were stopped and she was started on ivabradine at a dose of 0.025 mg/kg/day. Two hours after this first dose, she reverted to sinus rhythm (Figure 5). The dose of ivabradine was increased gradually to 0.1 mg/kg/day. Reversal from atrial to sinus rhythm was observed a few hours after each dose. The effect of ivabradine was sustained for around 4–6 h. Given the suboptimal response to ivabradine, ivabradine was stopped and amiodarone was started for rhythm control. Nonetheless, during this hospital stay, a mild improvement in LV systolic function was noted on echocardiography (see Table 3). Similarly, no side effects were attributed to the use of ivabradine. Her blood pressure remained within normal range, and she had no bradycardia or QT interval prolongation.

**Table 3:** Patients characteristics and their response to Ivabradine.

	**Case 1**	**Case 2**	**Case 3**
Gender	Female	Male	Female
Age at initial diagnosis	1 year 10 months	19 days	2 years
Age at which Ivabradine was received	2 years 6 months	19 days	9 years
Antiarrhythmics used before Ivabradine	Adenosine Metoprolol Amiodarone Flecainide	Amiodarone	Carvedilol Digoxin
Antiarrhythmics used in addition to Ivabradine	None	Amiodarone	None
Ivabradine dosage (mg/kg/day)	0.15	0.075	0.1
Outcomes induced by ivabradine	Suppression of supraventricular tachycardia Successful reversal to sinus rhythm Complete recovery of cardiac function	Suppression of atrial tachycardia Successful reversal to sinus rhythm	Rate control Mild improvement in LV function
Co-existing compromised LV function	Yes	No	Yes
% improvement in LVEF	30%	<5%	10%
Side effects attributed to Ivabradine	None	None	None
Success of ivabradine	Complete	Complete	Partial

The patient cardiomyopathy was believed to be secondary to her incessant atrial tachyarrhythmia. Optimal control of rate and rhythm through medical or interventional therapies is the mainstay of DCM management in this case.

## What is Known About Ivabradine

### Use of Ivabradine in Adult Patients

The clinical use of Ivabradine has been heavily investigated among adults with either CAD or chronic HF. In view of this, several randomized clinical trials (RCTs) were done to reveal the exact effect of ivabradine on the life quality of CAD and HF patients (33–42). The BEAUTIFUL trial was the first to examine the efficacy of ivabradine in these patients. It constitutes a multinational RCT in which 10,917 patients with CAD and a LV ejection fraction below 40% were enrolled (34). A significant drop in hospitalization for fatal and non-fatal MI, as well as a significantly reduced need for revascularization were reported among patients with a resting HR > 70 bpm (34, 43). However, in this first study, no significant improvement in mortality and HF hospitalization were noted in the ivabradine group, as compared to placebo (34). This study is an adequately blinded, controlled, and randomized clinical trial. The patients' medical regimens were continued regardless of the added medical intervention. Eighty-seven percent of these patients were maintained on β-blockers. This means that ivabradine can be safely co-administered with these medicines. However, the study is limited by the lack of additional therapeutic arms such as amiodarone and Class IC anti-arrhythmics. In this study, ivabradine was compared only to placebo.

The SHIFT trial was the second to discuss the influence of ivabradine on heart failure death and complications. It showed a significant reduction in cardiovascular death, and hospitalization for either HF or non-fatal MI particularly in ivabradine-treated patients with a baseline HR of 70 bpm and above (36). Ivabradine adverse effects were tolerated well and limited to bradycardia and visual symptoms (phosphenes) experienced by 5% and 3% of the therapy group, respectively (36). The incidence of serious cardiovascular, infectious and neurologic adverse effects was significantly higher in the placebo group. In this trial, the selection of the diseased cohort is considered adequate. Patients with a LV ejection fraction of more than 35% were excluded. Asymptomatic HF patients were likely excluded. Only patients with moderate to severe HF were included. HF medications, including β-blockers, were resumed in all patients. Nonetheless, just like the previous study, this trial is likely limited by the lack of additional treatment arms. Additionally, the exclusion of patients with HR <70 bpm, between 60 and 70 bpm, has hindered the evaluation of ivabradine efficacy in this particular group.

Furthermore, the SIGNIFY trial, published in 2014, studied the effect of ivabradine initiation in patients with isolated stable CAD having no clinical HF (38). It revealed no significant improvement in both fatal and non-fatal cardiovascular outcomes after 27.8 months of treatment (38). In this study, ivabradine use was associated with a significantly increased risk of bradycardia, atrial fibrillation, QT prolongation and phosphenes (38). These adverse events were seen in 7.9, 5.3, 1.8, and 5.4% of the participants, respectively (38). This suggests that ivabradine should not be added to the medical care of CAD patients in the absence of symptomatic HF. Indeed, owed to the lack of benefit in this group and to the reported potential side effects of ivabradine, ivabradine is currently only approved for patients with a resting HR of 70 bpm and above and is preserved for the treatment of symptomatic HF patients (4, 11, 12, 14, 44, 45).

Moreover, in one early study (INITIATIVE trial) published in 2005, ivabradine was found equally effective to atenolol in treating stable angina (33). The use of ivabradine in combination with atenolol for stable angina was later studied and found to be superior to atenolol plus placebo, in a separate study (35). Similarly, several studies have highlighted the benefit of ivabradine coadministration with β-blockers to patients with HF and suppressed LV ejection fraction (39, 42). In fact, the addition of ivabradine to standard therapies was linked to reduced HF mortality and hospitalization (39, 42).

Table 4 provides a synoptic comprehensive comparison of the above-mentioned clinical trials.

**Table 4:** A comprehensive comparison of the adult studies (BEAUTIFUL, SHIFT, and SIGNIFY trials).

**Trial**	**Number of enrolled patients**	**Characteristics of enrolled patients**	**Assessed outcomes**	**Findings**
BEAUTIFUL (34)	10,917	Patients with CAD and LVEF of less than 40%	1. Death 2. Admission to hospital for acute MI 3. Admission to hospital for HF	• Ivabradine exerted a significant improvement in **outcome 2** in patients with a resting HR of at least 70 bpm. • Ivabradine induced no significant improvement in **outcomes 1 and 3**.
SHIFT (40)	6,558	Patients with symptomatic HF, LVEF of 35% and less, in sinus rhythm with a HR of 70 bpm and above	1. Death 2. Admission to hospital for worsening HF	• The use of ivabradine led to a significant improvement in **outcomes 1 and 2**. • More serious side effects were experience by the placebo group. • Bradycardia and visual symptoms were experienced by 150 (5%) and 89 (3%) patients treated with ivabradine, respectively.
SIGNIFY (38)	19,102	Patients with stable CAD and with no HF and a HR of 70 bpm and above	1. Death 2. Incidence of non-fatal MI	• As compared to placebo, ivabradine was not associated with a significant improvement in **outcomes 1 and 2**. • The ivabradine group had a higher incidence of bradycardia.

*Ivabradine was compared to placebo in the above-mentioned cohorts of patients*.

### Use of Ivabradine in Pediatric Patients

#### Ivabradine and Focal Atrial Tachycardia

Focal atrial tachycardia (FAT) is a rare cause of tachyarrhythmia, representing up to around 15% of all supraventricular tachycardias (SVTs) (46, 47). It is very often an asymptomatic disease that affects both genders equally (46). FAT may rarely present with clinical symptoms such as palpitation, chest pain, shortness of breath, fatigue, lightheadedness and syncope (46). On the contrary, pediatric FAT often manifests with gastrointestinal and respiratory problems that include feeding difficulties, vomiting and rapid breathing (46). Symptoms may present at any age, mostly between 10 and 39 years of age (46).

FAT is rarely cured with standard antiarrhythmic medications such as β-blockers, Ca^2+^-channel blockers, class IC (i.e., flecainide) and class III antiarrhythmics (i.e., amiodarone) (48, 49). To date, the sole effective treatment of FAT is catheter ablation, however, it may not be applicable to all patients particularly in acute settings (17, 47, 50). FAT may prompt the development of serious morbidities such as atrial fibrillation and atrial flutter (47). It may also predispose, when chronic, to tachycardia-induced cardiomyopathy and heart failure (46, 47). FAT is believed to be caused by 3 main mechanisms that include: (1) abnormal automaticity, (2) micro-reentry, or (3) triggered activity (46, 47).

The use of ivabradine in the management of focal atrial tachycardia (FAT), refractory to conventional therapies, was grasped by a few case reports and case series, and also by a 28-patients prospective study (17–19). In this prospective study, ivabradine was found effective in reversing/suppressing FAT in 64% of the patients (18/28): 17 patients exhibited complete reversal to sinus rhythm and one patient had heart rate reduction from 152 to 74 bpm, after receiving an adequate first dose of ivabradine (17). The mean age of the enrolled patients was 34.5 years with a female predominance of 60.7%. Additionally, out of the 18 responders, 7 patients were pediatric aged 1 month, 7, 9, 14, 16, 16, and 17 years, respectively (17). The promissory efficacy of ivabradine, among these patients, has proven the implication of the funny current channels in the pathogenesis of FAT, particularly those originating from the atrial appendages (17). This further elucidates the role of abnormal automaticity in the generation of FAT.

In the pediatric population, a case report, published in 2011, has described the use of ivabradine in treating a 15 year-old female with FAT and secondary cardiomyopathy (18). The patient's tachyarrhythmia was resistant to a 3 month trial of β-blockers and amiodarone, however, it responded significantly to a 5 mg daily dose of ivabradine (18). Additionally, rate reduction from 150 to 90 bpm was noted on the second day of treatment. This was followed by complete resolution of symptoms and marked improvement of LV function. A 20% increase in LV ejection fraction (from 40 to 60%), was observed after 1 month of treatment (18).

Similarly, in a second case report, published in 2015, ivabradine was employed as a bridge to radiofrequency ablation in an 18 year old girl with left-sided FAT refractory to both medical therapy (adenosine, verapamil, flecainide, amiodarone, and atenolol) and electrical cardioversion (19). She received a daily dose of 10 mg and improved markedly within 5 h of ivabradine initiation. Radiofrequency ablation was performed after 2 days of ivabradine-mediated rate control (19). Six month later, she exhibited complete reversal to sinus rhythm and recovery of LV function (19).

Moreover, Janson et al. have discussed the successful use of ivabradine in two patients with focal ectopic atrial tachycardia aged 11 and 26 years, respectively (31). They attributed the efficacy of ivabradine to the enhanced automaticity suspected in cases of FAT. Ivabradine may suppress this automaticity resulting in rate control and effective reversal to sinus rhythm. Ivabradine's use in treating pediatric FAT has been likely portrayed in two case reports (30, 32). One case, of a 12-month-old girl with refractory ectopic atrial tachycardia, was safely and effectively managed with a combination therapy of ivabradine and metoprolol. No adverse events were encountered, and a sinus rhythm was attained after 2 days of treatment (32). The second case was a 5-month-old infant with multifocal atrial tachycardia stabilized and reverted to single ectopic atrial tachycardia with ivabradine. This infant was then offered permanent treatment using catheter ablation (30). Ivabradine was used in this case to overcome the multifocality of this patient's atrial tachycardia and to allow successful ablation.

#### Ivabradine and Junctional Ectopic Tachycardia

Junctional Ectopic Tachycardia (JET) is an uncommon type of SVT (51). JET may be a primary idiopathic disorder that manifests at birth or a secondary post-operative disorder that occurs following surgical correction of congenital heart disease (51). Primary JET, or congenital JET, is less common than post-operative JET (51). It represents a long-lasting illness that often manifests before the age of 6 months (52). Congenital JET may result in life-threatening cardiovascular complications such as cardiomyopathy, HF and ventricular fibrillation. It is likely associated with high mortality (51, 52). On the contrary, post-operative JET is often a transient disorder that exhibits a less-complicated clinical course (51).

Medical therapy is indicated in symptomatic patients with JET and in patients with compromised LV systolic function or tachycardic HR of 150 bpm and above (53). Medical therapy includes pharmacological and non-pharmacological interventions. Significant rates of therapy failure have been associated with pharmacological therapies such as amiodarone, digoxin, flecainide, propafenone, and propranolol (52). Permanent treatment of JET can be achieved only through catheter ablation (52).

The lack of appropriately effective pharmacological treatment has prompted the use of off-label medicines such as ivabradine. Nevertheless, this use was only discussed in a few studies. The efficacy of this medication was not evaluated by high-quality multi-center RCTs.

In one study, Al Ghamdi et al. have described a case of a 3 year-old-female with congenital JET treated successfully with ivabradine (20). The patient was diagnosed with congenital JET at the age of 27 days. At the time, she was given several medical therapies including amiodarone, β-blocker, procainamide, flecainide and sotalol. No reversal to sinus rhythm was achieved with any of these medicines (20). Only a moderate reduction in HR was attained with a combination of sotalol and flecainide. The patient was then discharged on this therapy. Nevertheless, only rate-control was achieved in this patient (20). Two years later, the patient presented with a JET of 160 bpm. Ivabradine was provided as a last resort before catheter ablation. After two daily dosages of 2.5 mg, she successfully reverted to sinus rhythm for the first time. Owed to ivabradine, catheter ablation was avoided in this patient and favorable outcomes were revealed on Holter monitoring (20).

In a second study, Dieks et al. have recommended the use of a combination of ivabradine and amiodarone in pediatric patients with congenital JET (21). They based their recommendation on the outcomes achieved using this combination in a group of 5 patients. Indeed, they reported the use of this combination in five patients aged between 10 days and 3.5 years (21). Ivabradine was initiated at a dosage of 0.05 to 0.1 mg/kg/day and was increased gradually to up to 0.28 mg/kg/day as needed. All of the patients were already maintained on amiodarone at a dose ranging between 5 and 10 mg/kg/day. Two patients were also maintained on propranolol. Additionally, One patient was maintained on digoxin and flecainide as well (21). After the first dose of ivabradine, rate control was achieved in all patients. However, reversion to sinus rhythm was noted in only three patients. In one patient, sinus rhythm was detected after 3 months of treatment (21). In the remaining patient, rate control and hemodynamic improvement were achieved but not rhythm control. Improvement in LV function was noted in all patients with defective function prior to treatment initiation. No Side effects, including bradycardia and hypotension, were encountered in any of the patients (21).

Despite the limited number of studied patients, this study provides strong evidence. It is the first case series that supports the off-label utilization of ivabradine in patients with treatment-resistant tachycardia such as congenital JET. However, ivabradine was not employed as a single therapy in this study. As a result, the use of ivabradine as a single therapy, without amiodarone, is not endorsed by this study.

Besides the above-mentioned studies conducted by Al Ghamdi et al. and Dieks et al., five additional studies have reported the use of ivabradine in treating JET. Kumar et al. have described two cases of post-operative JET successfully treated with ivabradine (22). First, they reported a case of JET in an 8 month-old male that developed after surgical repair of Tetralogy of Fallot. The patient was treated with amiodarone and esmolol but no rate or rhythm control was achieved (22). The patient was then offered a trial of ivabradine at a dose of 0.1 mg/kg/day. JET suppression and reversal to sinus rhythm were achieved after 2days of treatment. Second, they described a similar case of post-operative JET that developed in a 7 month-old male following surgical correction of ventricular septal defect. Ivabradine enabled successful reversal to sinus rhythm after 24 h (22). Nonetheless, in this study, they did not clarify whether ivabradine was used as a single or adjunctive treatment. As a result, the efficacy of ivabradine, as a single therapy, cannot be based on this study as well. Higher-quality studies are needed to endorse the efficacy of single ivabradine therapy in patients with JET.

In addition, Ergul et al. have suggested the use of ivabradine, as an adjunctive medicine, in patients with refractory congenital JET (23). They reported three cases of congenital JET with secondary cardiomyopathy and compromised LV function. These cases were treated with ivabradine and satisfactory outcomes were achieved after 24 h of treatment (23). Partial reversal to sinus rhythm was achieved in one patient. A complete suppression of JET and reversal to sinus rhythm was witnessed in the two other patients. Similarly, an improvement in cardiac function was attained in the three patients. Ivabradine was used at a dosage of 0.1 to 0.2 mg/kg/day (23). The three patients were treated with multiple antiarrhythmics but no improvement was noted. Ivabradine was added to the medical regimens of the patients. It was given as an adjunctive therapy combined with conventional antiarrhythmics such as amiodarone, digoxin, flecainide, and propranolol. No side effects were encountered in any of the patients (23). This study reports promissory findings, however, just the previous study conducted by Dieks et al., it has several limitations. In fact, the study is limited by (1) the small number of patients, (2) the short duration of follow-up, and (3) the lack of adequate assessment of ivabradine efficacy since it wasn't used as a single treatment in any of the patients.

Furthermore, two retrospective studies, published in 2019, have tackled the use of ivabradine in treating post-operative pediatric JET (27, 28). First, Krishna et al. reported an adequate response to ivabradine in a cohort of eight patients with JET acquired after open-heart surgery. The patients were aged between 3 days and 5 years. They were all successfully treated with a single therapy of ivabradine except one patient in whom reversal to sinus rhythm was achieved after the addition of amiodarone. Overall, an initial response to ivabradine, reflected by rate control, was attained within 160 min of treatment initiation in all patients. A longer duration of 3–16 h was required for complete resolution of JET and reversal to sinus rhythm (27). Second, Kumar et al. conducted a retrospective review of the charts of congenital heart disease patients admitted for cardiac surgery over 1 year. Only 20 patients (of 480) had acquired JET post-operatively, five of them had refractory JET and were adequately treated with ivabradine and amiodarone (28). Together, Krishna et al. and Kumar et al. supported the use of ivabradine, as a single or adjunctive therapy, in treating refractory post-operative JET.

#### Ivabradine and Other Tachyarrhythmias

The off-label use of ivabradine in treating other tachyarrhythmias has been also described in a couple of studies (24, 25). Romeo et al. reported a case of a 16 year-old male with treatment-resistant inappropriate sinus tachycardia and secondary cardiomyopathy. The patient was managed with several anti-arrhythmics: atenolol, digoxin, diltiazem, flecainide, and verapamil (24). Nonetheless, no adequate cure was achieved. Medical cardioversion with amiodarone and electrical cardioversion were likely ineffective. The patient condition was complicated by LV dilatation and reduced systolic function (24). Catheter ablation was considered the last resort and ivabradine was the last medical option in this patient. Patient was started on a 5 mg daily dose of ivabradine. The dose was increased gradually to 15 mg/day (24). Normal sinus rhythm and proper ventricular dimensions and function were achieved after 3 months of treatment with ivabradine. No additional medications were used and no adverse events attributed to ivabradine were witnessed (24).

Furthermore, in a retrospective observational study published in 2017, safety and efficacy of ivabradine was assessed in a cohort of 22 patients with postural orthostatic tachycardia syndrome (POTS) (25). Ivabradine was effective in 15 patients in whom proper HR reduction was achieved with a dose of 0.2 mg/kg/day. No improvement, and no worsening of symptoms, was noted in 6 patients (25). However, clinical deterioration, despite treatment with ivabradine, was observed in one patient. No cardiac side effects, such as bradycardia and QT interval prolongation, were reported. Only one patient developed phosphenes that was reversed by dosage reduction (25). This study was the first to report the safety and efficacy of ivabradine in a cohort of patients with POTS. However, the study was limited by (1) the small number of participants, (2) the lack of randomization, blinding and control, and (3) the lack of adequate assessment of ivabradine sustained efficacy through 24-h Holter monitoring.

#### Ivabradine in Children With DCM and Symptomatic HF

The efficacy of ivabradine in improving clinical status of children with DCM and symptomatic HF was evaluated by a single randomized placebo-controlled clinical trial published in 2017 (26). In this study, 116 children with DCM and HF were assigned to one of two treatment groups: ivabradine and placebo (26). Over a 1 year period of follow-up, LV systolic function, clinical status, quality of life and the level of N-terminal pro-BNP were evaluated and compared between the two treated groups. A significant improvement in LV systolic function was detected in the ivabradine group (26). Similarly, both clinical status and quality of life were improved in the ivabradine group, however, this improvement was not significant. No difference in N-terminal pro-BNP levels was noted. Additionally, the frequency of adverse effects was identical in both groups (26).

Overall, favorable outcomes were achieved with the use of ivabradine in these patients. The study is a high-quality RCT that is well-blinded and controlled. However, it is limited by the reduced number of treated patients, the limited number of assessed outcomes, and the inability to prove improved mortality in patients treated with ivabradine owed to the small number of patients and the short duration of follow-up.

## Discussion

Increased HR is associated with increased myocardial oxygen demand and compromised diastolic perfusion. Hence, appropriate control of HR is absolutely needed to prevent fatal complications such as myocardial ischemia. Congruently, maintaining a well-controlled average HR is essential to enhancing myocardial oxygen supply and thus to maintaining a favorable ratio of myocardial oxygen demand to supply. Similarly, HR control in patients with tachyarrhythmias is crucial for the prevention of secondary cardiovascular complications such as DCM and symptomatic HF.

As mentioned previously, evidence derived from adult studies has supported the use of ivabradine in CAD and HF patients with a resting HR of at least 70 bpm. However, ivabradine was proven less effective in improving HF-associated morbidity and mortality in adult patients with a resting HR of <70 bpm. This is attributed to the use-dependence property of ivabradine that makes ivabradine more effective in patients with high-normal to elevated HR. This means in turn that ivabradine should be solely provided to patients with a resting HR of 70 bpm and above to prevent unnecessary interventions and also to hamper the occurrence of undesired side effects such as bradycardia and QT interval prolongation.

**Figure 6:**
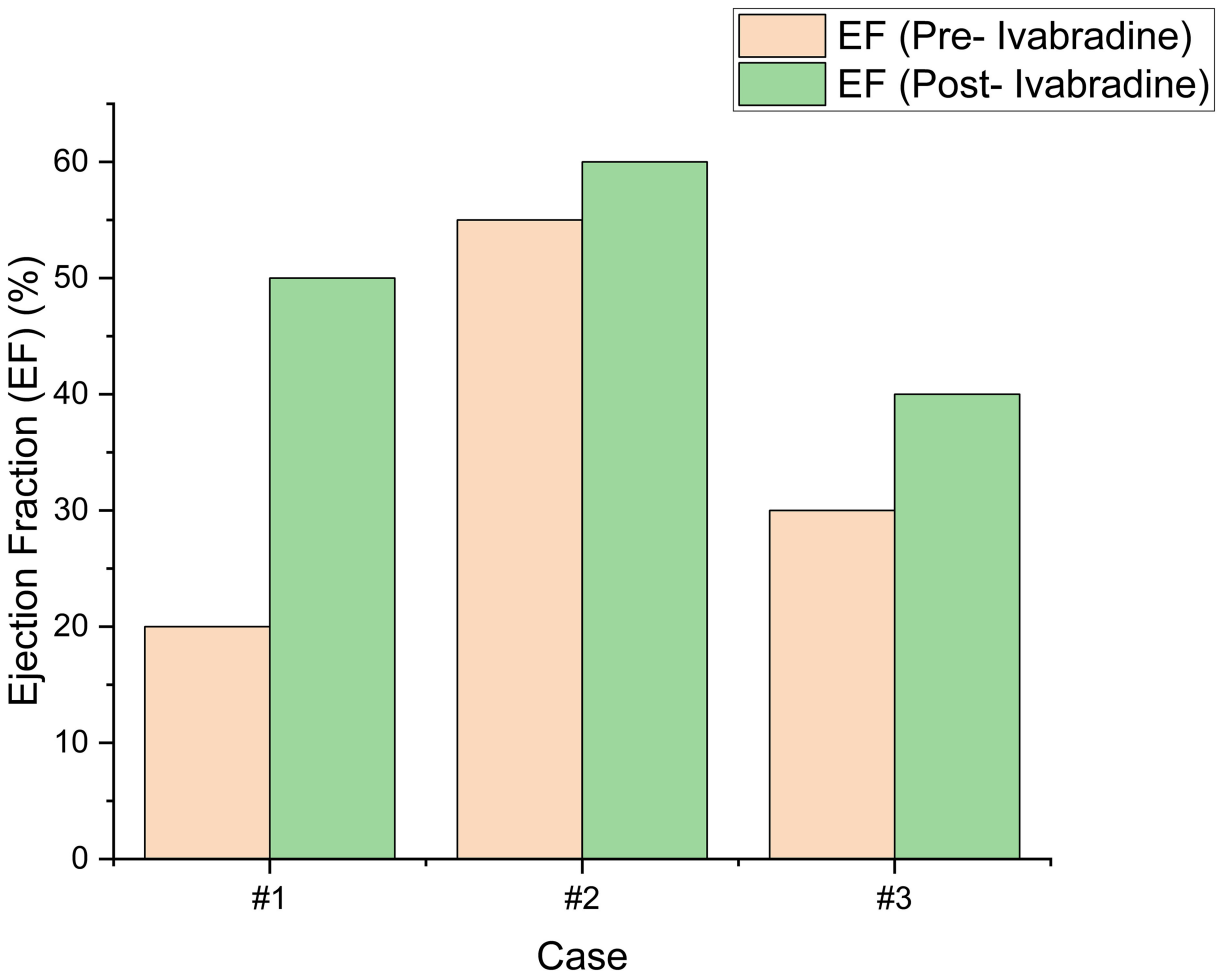
The left ventricular ejection fraction (LVEF) of our patients pre- and post-ivabradine. A significant improvement of 30% was achieved in our first patient. On the contrary, a modest improvement (of 10%) in LVEF was noted in patient #3. To note, our second patient had no LV systolic dysfunction prior to ivabradine intake. His LVEF remained within normal thereafter and improved by around 5%.

The lack of well-supported high-quality evidence in the pediatric population has limited the use of ivabradine in this population. This has led in turn to the limited number of studies reporting the utilization of ivabradine in children. As mentioned previously, around a dozen studies has discussed this off-label use in pediatric patients with tachyarrhythmias or symptomatic HF. Here, in our study, we report three cases of SVT treated with ivabradine as a solo or adjunctive therapy employed in addition to other conventional anti-arrhythmics. Favorable outcomes, denoted by rate control, reversal to sinus rhythm and improved LV function (Figure 6), were achieved in two of our patients (patients number 1 and 2). However, only rate control and a modest improvement in LV function were achieved in patient number 3. As a result, ivabradine was discontinued in this patient and amiodarone was initiated for rhythm control. The patients were aged 2 years and a half, 19 days, and 9 years, respectively.

Treatment-resistant atrial tachycardia prompted ivabradine initiation in two of our patients. However, unlike patient number 3 who received ivabradine as a single therapy, patient number 2 was treated with a combined therapy of amiodarone and ivabradine. This can explain the superior outcomes obtained in this patient. Additionally, this is congruent with the results reported by Dieks et al. As mentioned previously, Dieks et al. have endorsed the effectiveness of ivabradine and amiodarone in treating congenital JET (21). In their study, reversal to sinus rhythm and suppression of congenital JET were attained in 80% of the patients (4/5). Adequate HR control with no reversal to sinus rhythm was achieved in the remaining patient. In fact, partial treatment failure was encountered in a 2 year-old female who had the most resistant congenital JET among the treated patients. This patient was treated with digoxin and flecainide in addition to amiodarone and ivabradine, and yet no adequate response was detected (8). Similarly, our third patient was likely known to have an extremely resistant atrial tachycardia that failed several medications. Subsequently, we conclude that a favorable clinical outcome could have been achieved in this patient by the addition of amiodarone to the ivabradine regimen. Additionally, it seems that patients with chronic and multi-treatment resistant SVT, like this patient, are less likely to respond successfully to ivabradine. Treatment naïve SVTs are more responsive to ivabradine. Moreover, promissory clinical outcomes can be associated with early initiation of ivabradine in patients with SVT.

The daily dosages of ivabradine received by our patients have ranged between 0.025 and 0.15 mg/kg. The dosing regimen was extrapolated from doses used in treating adult patients with angina or tachycardia at our center. A twice-daily dosage of 5 to 7.5 mg is often prescribed to adult patients requiring ivabradine. Hence, we agreed on starting our pediatric patients on a dosage of 0.05 mg/kg/day and on increasing this dosage gradually, if needed, to up to 0.15 mg/kg/day. Our team was markedly cautious since ivabradine is not yet approved for use in pediatric patients with refractory SVT. Additionally, our medical center abides firmly by the American guidelines and imposes strict regulations on using off-label medications, this prevented us from using higher doses of ivabradine in our pediatric population. The patients' parents were also educated and informed about the side effects that may result from this off-label use of ivabradine. Furthermore, we noted that similar dosages were used by most of the above-mentioned studies. However, in the study performed by Dieks et al. and Ergul et al., the dosage of ivabradine was boosted up to 0.28 and 0.2 mg/kg/day in some patients, respectively (21, 23). This can explain the suboptimal response achieved in our third patient who received a maximum dosage of 0.1 mg/kg/day. Congruently, a higher dosage could have resulted in a more effective response in this patient. Nonetheless, further studies are needed to determine the acceptable maximal dosage of ivabradine that can be safely used in children with tachyarrhythmias.

Equal to most of the above-mentioned studies, no side effects attributed to ivabradine were experienced by our patients. However, in one study involving 22 patients with POTS, phosphenes was experienced only by one patient (25). No cardiovascular side effects, such as hypotension, bradycardia, or QT interval prolongation, were encountered neither in our study nor in the above-mentioned studies. In sum, the off-label use of ivabradine in pediatric patients is supported by the lack of frequent and serious adverse effects and thus by the well-tolerated safety profile of ivabradine.

Furthermore, treating refractory SVT with ivabradine may successfully prevent the need for costly interventions such as radiofrequency catheter ablation or catheter cryoablation. Interestingly, the use of ivabradine in these patients can aid in providing satisfactory clinical outcomes at lower costs. In fact, the cost of one ablation can cover for the treatment of at least a dozen of patients with ivabradine. This is of utmost importance particularly in low- to middle-income countries, such as Lebanon, where proper healthcare services and effective medical interventions are not easily accessible to all citizens. Additionally, the use of ivabradine instead of catheter ablation can prevent the occurrence of undesired side effects that may be associated with catheter ablation such as bleeding, post-operative infection, iatrogenic atrioventricular block, and valvular injury.

Our study is the first to report the off-label use of ivabradine in treating pediatric SVT in the Middle East and North Africa region. It is also the first to report the use of ivabradine as a single therapy in pediatric patients with treatment-resistant SVT. It highlights the benefit induced by this novel medication in these patients. Additionally, it provides a thorough and up to date review of the available evidence. However, the study is limited by the small number of patients and the lack of prolonged follow up and long-term monitoring of clinical outcomes in these patients. This limits our ability to demonstrate moderate- and long-term efficacy and safety of ivabradine.

## Conclusion

Ivabradine is a novel medication that is well-tolerated and effective in improving the clinical status of adults with symptomatic HF. The medication is not yet approved for use in pediatric patients with tachyarrhythmias. However, promissory outcomes have been associated with the off-label use of ivabradine in treating pediatric SVT. Herein, based on evidence deriving from the above-mentioned studies and from our experience at the Children's Heart Center at the American University of Beirut Medical Center (AUBMC), we suggest the utilization of this safe, inexpensive and effective medicine in treating children with refractory SVT. Additionally, we encourage the establishment of high-quality controlled randomized clinical trials that assess the efficacy of ivabradine in this population, and compare its efficacy to other well-known and approved antiarrhythmics.

## Data Availability Statement

The raw data supporting the conclusions of this article will be made available by the authors, without undue reservation.

## Ethics Statement

The studies involving human participants were reviewed and approved by The American University of Beirut - Institutional Review Board. Written informed consent to participate in this study was provided by the participants' legal guardian/next of kin. Written informed consent was obtained from the minor(s)' legal guardian/next of kin for the publication of any potentially identifiable images or data included in this article.

## Author Contributions

MA developed the idea and the study framework. BA-S, FA, OE, CT, MA, and FB attended to the patients and followed up on their care in the Children's Heart Center at The American University of Beirut. NY wrote the first draft of the manuscript, and performed data collection and analysis, and literature review. All authors contributed to corrections and adjustment of subsequent iterations of the manuscript and approve and agree with the content.

## Conflict of Interest

The authors declare that the research was conducted in the absence of any commercial or financial relationships that could be construed as a potential conflict of interest.

## Publisher's Note

All claims expressed in this article are solely those of the authors and do not necessarily represent those of their affiliated organizations, or those of the publisher, the editors and the reviewers. Any product that may be evaluated in this article, or claim that may be made by its manufacturer, is not guaranteed or endorsed by the publisher.
